# Ancient inversion polymorphisms associate with sexually selected traits across natural guppy populations

**DOI:** 10.1371/journal.pbio.3004024

**Published:** 2026-09-23

**Authors:** Yuying Lin, Wouter van der Bijl, Judith E. Mank

**Affiliations:** Department of Zoology and Biodiversity Research Centre, University of British Columbia, Vancouver, BC, Canada; University of Cambridge, UNITED KINGDOM OF GREAT BRITAIN AND NORTHERN IRELAND

## Abstract

Understanding the distribution, frequency, and long-term persistence of chromosomal inversions in natural populations is key to understanding population evolution and adaptive processes. Inversions often span multiple genes, are subject to strong selective pressures and can affect complex traits. Here, we used an unbiased method to identify inversions in guppies from three different Trinidadian rivers with paired high- and low-predation populations which differ in a wide range of morphological, behavioural and life-history traits. We identified 22 inversions, ranging in age from 1 to 6 million years, which are widespread across the genome and predate the colonisation of each river. We were able to verify the breakpoints for all but one of these inversions using linked reads. We find three inversions that are significantly associated with local adaptation syndromes in high- versus low-predation populations, however, none are reciprocally fixed throughout all three replicate rivers. Additionally, we observe seven additional inversions maintained in all three rivers without evidence of local adaptation. Simulations reveal that the level of inversion polymorphism that we observe is far greater than expected under a neutral model. We observed a significant overlap between polymorphic inversions and loci previously implicated in male ornament pattern variation in guppies, suggesting that negative frequency-dependent selection due to female preference for male pattern novelty explains the maintenance of inversion polymorphisms. Overall, our results show the role of sexual selection in the long-term maintenance of inversion polymorphisms, and an interplay between sexual and natural selection in frequency dynamics.

## Abstract 2

Genomic inversions spanning many genes can persist for millions of years. This study of Trinidadian guppy populations reveals how both ecological conditions and mate choice help maintain this hidden layer of genomic diversity, linking sexual selection with long-term genome evolution.

## Introduction

Chromosomal inversions represent a significant source of genetic variation, often spanning multiple genes and affecting a wide range of complex traits [1]. In some cases, recombination is not possible in inversion heterozygotes, however, in those cases where it is still possible, it results in unbalanced and non-viable gametes [2] and recombinants are selected against. Therefore, inversions potentially reduce cross-overs, suppress recombination and preserve locally adapted allelic combination [3].

The evolutionary fate of inversions depends on the interplay between selection, drift, and gene flow [4,5]. Theoretically, when a new inversion arises in a population, it is either eventually fixed or lost if there are no countervailing forces [4]. Accordingly, inversions have been shown to be associated with diverse adaptative traits across animals and plants, including butterfly wing colouration [6], ecological adaptation in deer mice [7], migration trajectory [8] and reproductive strategies in birds [9] and eco-type divergence in sunflowers [10,11]. In cases associated with local adaptation, inversions may be fixed by selection within populations but vary across the broader species distribution. Alternatively, recent empirical studies suggest that some inversion polymorphisms can be maintained within populations by long-term balancing selection [5,12–14].

Guppies (*Poecilia reticulata*) are a well-established model of adaptation in response to both natural [15–17] and artificial selection [18–20]. Following the colonisation of Trinidad, likely in the Pleistocene [21], the Eastern (Oropouche, including the Quare River) and Western (Caroni, including the Aripo River) drainages became separated by a watershed divide roughly 600,000–1,000,000 years ago [21,22]. Before separation from mainland South America in the Holocene [23], the Eastern, Western and Northern Drainages (which includes the Yarra River) were likely all connected to the Orinoco River. Many rivers in Trinidad have distinct high- and low-predation populations that experience contrasting biotic (e.g., predation pressure, food availability) and abiotic (e.g., temperature) conditions, which affect key life-history traits such as body size, age at maturity and growth rate [16,24].

Genetic mechanisms underlying adaptation mainly include new mutations and standing genetic variation [25,26]. Although highly variable, average guppy mutation rates are similar to other vertebrates [27,28]. This, combined with the lack of evidence for strong selective sweeps expected with adaptive de novo mutations [29] suggests that standing genetic variation may be a major contributor to adaptation in guppies. However, convergent phenotypic adaptation has been associated with limited convergent genomic signatures expected if adaptation is due to large-effect alleles in guppies [17,29–31].

To better understand the role of inversions in guppy adaptation, we identified and characterised the frequency and distribution of inversions in wild-caught guppy adults collected from replicate upstream and downstream populations across three Trinidadian rivers. Our results suggest inversions are common in the guppy genome, and although they exhibit different genotype frequencies, are largely maintained as polymorphisms within and among populations at far higher levels than would be predicted based on neutral expectations. Most inversions we identified date prior to the separation of the major Trinidad drainages, and include loci implicated in male colouration variation, which are subject to negative frequency-dependent selection [32,33]. Overall, our study indicates that sexual selection can maintain inversion variation in natural populations over long evolutionary timescales.

## Materials and methods

### Collecting samples and data

Data from all samples are archived at NCBI under project number PRJEB39998. Fish were collected from three Trinidadian rivers, Aripo, Quare and Yarra, each of which includes one upstream population with low predation, and one downstream population with high predation pressure. Permits for sampling fish were obtained from Trinidad Ministry of Agriculture, Land and Fisheries and Indar Ramnarine. From each population, we collected 10 males and 10 females, each of which were sequenced individually to an average read depth of ~25× after quality control. Full details are in Almeida and colleagues [34].

### SNP calling and genotyping

First, we trimmed low-quality raw reads and adaptors with Trimmomatic v0.39 [35]. High-quality reads were then mapped to the female guppy reference genome [36] using BWA mem v0.7.15 [37]. BCFtools v1.16 [38] mpileup and call were employed to genotype samples, with only SNPs retained for further analysis. We then removed SNPs with (1) minor allele frequency <0.01, (2) >20% missing data, (3) <10× and >80× coverage with VCFtools v0.1.12b [39].

### Inferring population structure

To identify the population structure of samples we collected, we first converted the filtered genotype data to PLINK binary format using PLINK 2.0 [40]. Population structure was examined using PLINK PCA. Principal components (PCs) were computed from both genome-wide and sex chromosome (chromosome 12) genotype dataset to assess major axes of genetic variation. We pruned SNPs in linkage disequilibrium (*r*^2^ > 0.8) to remove correlated markers using PLINK 2.0 before estimating ancestral population components were estimated using ADMIXTURE v1.3.0 [41]. We evaluated values of *K* = 1 through *K* = 8 to capture a range of possible population structures exceeding the expected number of populations, as suggested by previous studies [41–43]. Cross-validation (CV) error was computed for each *K*, and the *K* with the lowest CV error was considered optimal.

### Identifying structural variants

We used localPCA (lostruct) [44] to identify and characterise inversion polymorphism within each population, between each low-predation (upstream) and high-predation (downstream) population pair in each river, and across all samples respectively, with 100 SNP windows and default parameters. Focusing on MDS1, MDS2, where inversion signals are typically detected, as well as the consecutive differences in these two MDSs, we used a hidden Markov model (HMM, https://github.com/hmmlearn/hmmlearn) to identify outlier genomic regions exhibiting patterns of genetic differentiation that distinguish subsets of samples from the genomic background along each chromosome. We identified inversion boundaries, and assigned each 100 SNP window to an optimal number of clusters, determined by the elbow method, implemented within the HMM model. For each identified outlier region, we performed PCA with all SNP data on the entire region using scikit-allel [45], and genotyped samples from their clustering in the first principal component.

### Breakpoint verification

We used linked-reads (see [34] to validate each inversion we identified. We first mapped reads with barcodes to the female reference genome [36] using LongRanger wgs (https://github.com/10XGenomics/longranger), and then we used Loupe v2.12.2 from 10× Genomics to extract barcode sharing matrices for each inverted region and their flanking region, We visualised the barcode sharing across the identified inversions. To verify the barcode sharing enrichment in the inverted regions and to reduce artefacts associated with self-interactions, barcode sharing values along the main diagonal were masked. Specifically, for matrix elements within a fixed bandwidth (±10 bins) of the diagonal, we first excluded missing data, and then calculated summary statistics including mean, median, standard deviation, and maximum values across all elements. We also calculated these values for non-zero elements to account for sparsity in the matrices.

### Calculating linkage disequilibrium (LD) and decay

For each putative inversion, we calculated the LD on genotype data from all individuals with VCFtools v0.1.12b [39]. A 10 kb window size and 5 kb window step were applied when calculating LD following Todesco and colleagues [11]. We also used PopLDdecay v3.43 [46] to infer the genome-wide LD decay pattern in each population.

### Calculating genetic differentiation

To measure genetic differentiation between different inversion genotypes, we performed pairwise *F*_ST_ between predicted homozygote inversion genotypes, ref/ref (cluster 0) and alt/alt (cluster 2), clusters from PCA clustering using scikit-allel described in “Identifying structural variants”. We also measured inter-population *F*_ST_ between high- and low-predation populations in each river.

### Inferring demographic history

To infer demographic history of the samples, we first generated a whole-genome diploid consensus sequence using SAMtools and BCFtools and then used ‘vcfutils.pl vcf2fq -d 10 -D 80’ to remove SNPs with coverage that are not in the range between 10× and 80×. We ran PSMC v0.6.5 [47] with default parameters for each sample separately. For visualisation of the PSMC results, we used a mutation rate of 1.35 × 10^−^⁸ per site per generation [28] and a generation time of 210 days [48].

### Inferring age of inversions

We inferred age of each inversion following methods described in Hill and colleagues [49]. Briefly, we estimated the age in generations (*T*) of an inversion as


$$\begin{array}{c} T\;=\frac{d_{A}}{\text{2}\mu } \end{array}$$

where $d_{A}$ is the net genetic divergence between the two homozygous inversion genotypes and *µ* is the mutation rate (taken from [28]. The net genetic difference [50] was calculated as


$$\begin{array}{c} d_{A}\;=d_{xy}-\frac{\pi_{ref/ref}+\;\pi_{alt/alt}}{\text{2}} \end{array}$$

where $d_{xy}$ is the mean pairwise divergence between genotypes and $\pi_{ref/ref}$, $\pi_{alt/alt}$ are the within-genotype nucleotide diversities. We note that this age estimate is sensitive to the mutation rate, for example, using the mutation rate of 2.9 × 10^−^⁹ reported by Burda and Konczal [27] would result in estimate ages approximately four times older.

### Inversions under neutrality using forward genetic simulations

To evaluate whether the observed persistence of inversions across multiple rivers is compatible with neutral expectations from genetic drift, we conducted forward simulations using SLiM v5.1 [51]. We simulated three isolated populations, representing the three rivers after their split, each following the inferred demographic histories of the three rivers as estimated by PSMC analyses (details see 3.2.7). We used the lower estimate of the river divergence time, 600,000 years or 1 million generations [21,22]. To further ensure a conservative test, we combined the effective population size estimates across individuals by calculating their median on the log scale, and the high- and low-predation populations within each river were summed.

At generation 1, a single neutral mutation was introduced at frequency 0.5 in each population, corresponding to a scenario in which the inversion was already present at high frequency in the ancestral population, thereby maximising the opportunity for persistence under drift. Populations then evolved under a Wright–Fisher model with random mating, and the generation at which the mutation was no longer segregating (i.e., lost or fixed) in each population was recorded. To improve computational efficiency, we applied a standard rescaling factor *k*, dividing both population sizes and time by *k*, so that the ratio *t*/*2N*_*e*_ remains constant. Reported times were converted back to unscaled generations. We performed 1,000 runs at *k* = 20, and then confirmed that the conclusions held at different values of *k* by performing 250 runs at *k* = 10 and 100 runs at *k* = 5.

### Reconstructing phylogenetic tree across samples

To reconstruct the phylogenetic tree of inversions across all the samples, we mapped resequencing reads to the *P. reticulata* reference genome [36] using BWA mem [37] and genotyped across samples and with *P. picta* as an outgroup using BCFtools [38]. We used vcf2phylip (https://github.com/edgardomortiz/vcf2phylip) to convert SNP data into PHYLIP format with ambiguous characters encoded using IUPAC. We then used IQ-TREE3 [52], with both ModelFinder’s automated model selection and the GTR DNA substitution model to reconstruct the phylogenetic tree for each inversion. Inversion heterozygotes were excluded from the phylogenetic analysis. To infer the phylogenetic relationships among samples from different rivers, we used RAxML [53] to reconstruct a phylogenetic tree based on genome-wide SNP data, following the same pipeline described above.

### Association with environment

For each inversion, we tested its association with high- and low-predation environments across three parallel population pairs. We calculated genotype and haplotype frequencies for each population and assessed whether specific haplotypes were consistently more abundant in either high- or low-predation populations across rivers. For inversions showing consistent haplotype patterns across rivers, we used haplotype frequencies to perform a Cochran–Mantel–Haenszel (CMH) test and inversions with a CMH *p*-value <0.05 were considered significant.

### Inversions capturing loci under sexual selection

We searched for SNPs significantly associated with male orange and black colour variation (*p*_adj_ < 0.05), as inferred from the GWAS results reported by Van der Bijl and colleagues [20]. Sequence data are available via the SRA under accession PRJNA1262490 (http://www.ncbi.nlm.nih.gov/bioproject/1262490), and animal care protocols were approved by the University of British Columbia animal care committee (A22-0239). We first examined the tested for the presence of significant SNPs within inversions. We reduced significant SNPs to independent peaks by LD-clumping with an *r*^2^ cut-off of 0.2. Because inversions can generate long-range LD, we clumped across each whole chromosome rather than within a fixed physical window. We used two sets of SNPs, one associated with a male’s coordinates in a multivariate space of heritable pattern features (hereafter “pattern space”), and one comprising loci associated with the presence/absence of each of 12 individual sexual ornaments. We excluded Y-derived SNPs that mapped to the autosomes as identified in Van der Bijl and colleagues [20] and calculated the number of LD-clumped SNPs associated with pattern space and presence/absence in each inversion. To further verify the set of the GWAS loci are not derived from the Y chromosome, we calculated the male-to-female read depth ratio across each inversion. We additionally tested whether the inversions segregating in QuH, the wild source population of our GWAS panel, also segregate in the panel itself, by performing a PCA on each inverted region, 3-means clustering on PC1, and checking for elevated heterozygosity in the middle cluster.

To test whether the GWAS peaks overlap with inversion regions in a higher frequency than expected by chance, we performed a permutation test. First, we shuffled the position of those clumped SNPs using BEDTools shuffle [54], excluding non-callable genomic regions, defined as regions with zero read depth across all samples. Because chromosome 12 is the sex chromosome, where colour loci are disproportionally located, we excluded chromosome 12. Then, we counted the overlap for each shuffle using BEDTools intersect [54], repeated this process 100,000 times, and compared the observed overlap with the permuted null distribution. Finally, we performed an additional permutation test with 100,000 iterations to assess whether inversions are enriched in genic regions relative to null expectation.

To evaluate the extent to which these colour pattern loci contribute to phenotypic variation, we used the phenotypes and genotypes of 297 males from the artificial selection lines reported by Van der Bijl and colleagues [20]. For pattern space, we fitted linear models to each of the five embedding dimensions and quantified the variance explained using the coefficient of determination (*R*^2^). For ornament presence/absence, we fitted logistic regressions and used Nagelkerke’s *R*^2^. In both cases, we fitted three models: a baseline model, a model with only the loci which overlap an inversion, and a model with all loci associated with that trait. All three models included a fixed effect for selection regime. We report the phenotypic variance explained by inversion-overlapping loci as the difference between the baseline model and the model with inversion-overlapping loci, to remove inflation of effects due to selection. Since GWAS cannot detect all loci of effect, we additionally report what proportion of variance the inversion-overlapping loci explain compared to all trait-associated loci evaluated. Component estimates (i.e., the embedding dimensions within pattern space, and the individual ornaments within ornament presence) were combined into a single value by weighting them by their phenotypic variance.

### Functional inference

We identified genes inside inversions based on the genome annotation downloaded from Ensembl. We extracted genes in the inversions shared across all three rivers using BEDTools [54], and visualised the number of genes present in shared inversions with upset plots. We extracted Ensembl gene IDs from the genome annotation file and performed GO enrichment analysis using DAVID [55] with inversions significantly associated with high- and low-predation population differences inferred from “association with environment”.

## Results

### Population structure and demographic history

PCA analysis of the whole genome, including the sex chromosome (chromosome 12) shows six major clusters among individuals, corresponding to the six populations across the three rivers we sampled in Trinidad (S1 Fig). The first two PCs explain >75% genetic variation among populations, suggesting substantial population differentiation.

The mean population differentiation, as determined by *F*_ST_ values across the genome, between high- and low-predation populations was 0.1052 for the Aripo River (95% CI: 0.1046, 0.1059), 0.3372 for the Quare River (95% CI: 0.3360, 0.3385), and 0.3561 for the Yarra River (95% CI: 0.3549, 0.3573) (S2 Fig), which is qualitatively similar to the PCA results (S1A Fig), where some individuals from Aripo high-predation population (ArH) cluster together with the low-predation population (ArL), while the other two rivers each exhibit two distinct clusters. This pattern suggests there is limited detectible post-divergence gene flow across or within rivers. The genetic diversity is moderate in Trinidadian guppies, with genome-wide *π* values of 3.127 × 10^−3^ (95% CI: 3.115 × 10^−3^, 3.140 × 10^−3^) for Aripo, 3.289 × 10^−3^ (95% CI: 3.276 × 10^−3^, 3.301 × 10^−3^) for Quare and 3.516 × 10^−3^ (95% CI: 3.504 × 10^−3^, 3.528 × 10^−3^) for Yarra (S3 Fig).

Our PSMC results align with the vicariance history (S4 Fig) [21,22,56]. The PSMC-estimated effective population size in low-predation populations is slightly smaller than the high-predation populations, this is also consistent with the genome-wide LD decay pattern, with smaller effective population size exhibiting elevated overall linkage (S5 Fig).

### Inversion polymorphisms

Using a Hidden Markov Model (HMM) to identify inversion boundaries, we identified 22 inversions (Fig 1) distributed across the guppy genome. Using the inversion on chromosome 5 as an example (Fig 2), localPCA analysis suggests the 31.88–33.18 Mb region exhibits different population structure, compared to other genomic regions along the chromosome (Fig 2A) consistent with an inversion. The PCA for this region shows three distinct clusters, which represent reference homozygous, heterozygous, and alternative homozygous inversion genotypes (Fig 2B). Genetic differentiation (*F*_ST_) between homozygotes for the reference genotype (cluster 0) and the alternative genotype (cluster 2), is significantly higher than other chromosomal regions (Fig 2C). Heterozygotes for the inversion exhibited the highest level of SNP heterozygosity as expected (Fig 2D). The inversion exhibits elevated LD, compared to other regions from the same chromosome (Fig 2E). Genotype visualisation and phylogeny restricted to this region further support that the presence of an inversion where the same inversion genotype across rivers clustered together, with both automated model selection and GTR models yielding quantitatively similar phylogenies (Figs 2G, 2H, and S6). Other inversions are shown in S7–S27 Figs.

**Fig 1:**
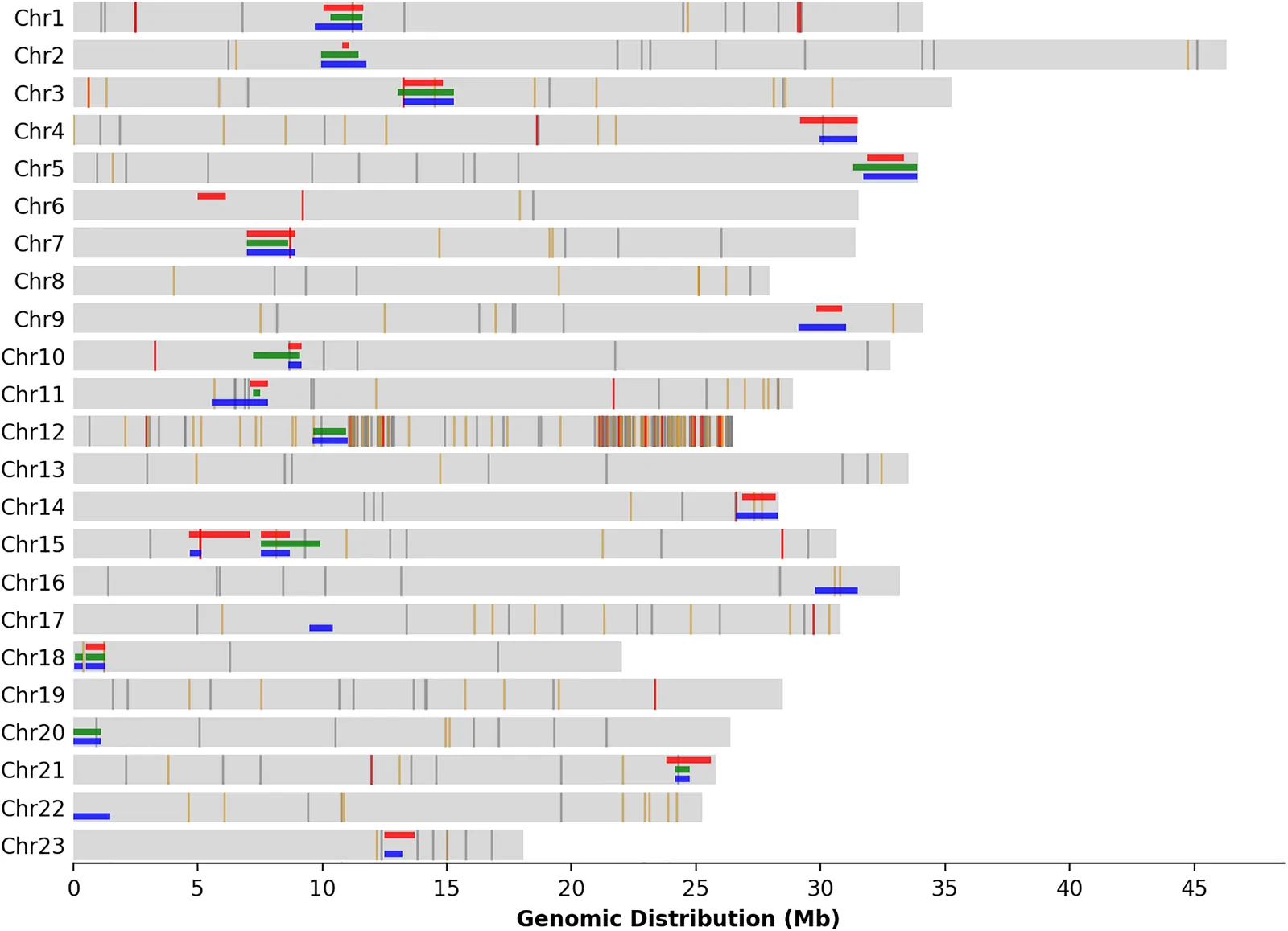
Genomic distribution of inversions. Location of inversions in Aripo (red), Quare (green) and Yarra (blue) rivers. The inversion boundaries were inferred using localPCA and HMM, see Materials and methods for details. Vertical bars (grey: present/absence of specific colour ornaments; orange: pattern space, red: both present/absence and pattern space) are significant GWAS SNPs (*p*_adj_ < 0.05) associated with colour and pattern variation in male guppies, from Van der Bijl and colleagues [20]. The data and code needed to generate this figure can be found at https://doi.org/10.5281/zenodo.22177863.

**Fig 2:**
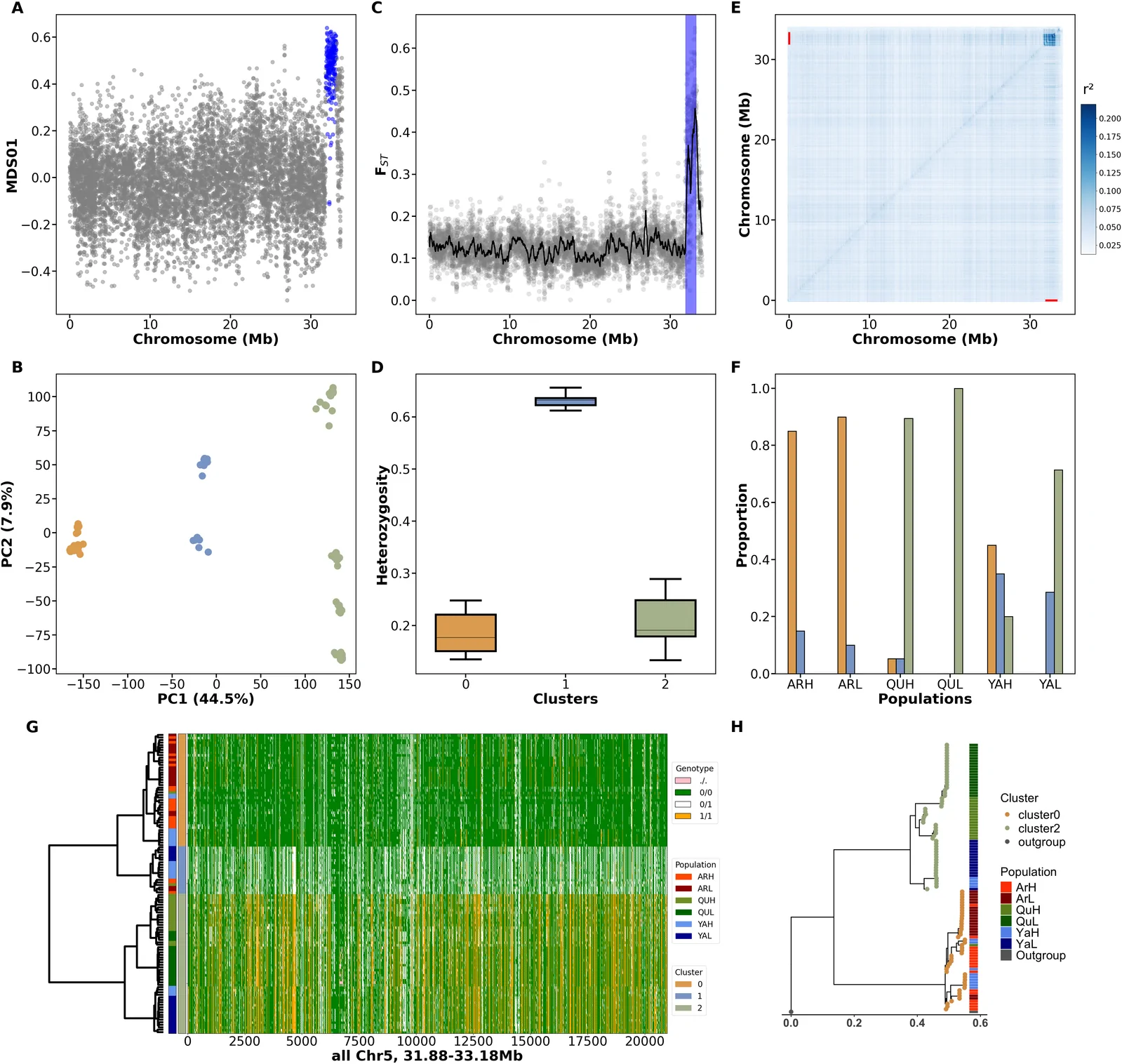
Identifying inversions. Using Chromosome 5, 31.88–33.18 Mb across rivers as an example. **A.** MDS01 along chromosome 5 with the inversion highlighted in blue. **B.** PCA of the inversion shows three distinct clusters, representing the reference homozygote, heterozygote, and alternative homozygote genotypes. **C.** *F*_ST_ between two homozygous inversion genotypes (cluster 0 and cluster 2). **D.** Average proportion of heterozygous SNPs across individuals in three clusters corresponding to panel B. **E.** LD plot. **F.** Proportion of three different inversion genotypes in high- and low-predation populations. NS: no population displays significant deviation from HWE. **G.** Genotype plot of all individuals in the focal population. Grey, homozygous reference alleles (0/0); orange, heterozygous alleles (0/1); green, homozygous alternative alleles (1/1); white, missing genotype (./.). *X* axis, SNPs; *Y* axis, sample names; Population colour bar, population colour corresponds to these in Fig 1; Cluster colour bar, corresponds to those in panel B. **H.** Phylogeny of inversions including individuals from cluster 0 and cluster 2. Cluster colour and population colour are the same as those in panel G. The data and code needed to generate this figure can be found at https://doi.org/10.5281/zenodo.22177863.

For each inversion, we used 10× Genomics linked reads [34] to validate breakpoints (S28 Fig and S1 Table). We were able to validate all but one of our identified inversions, and the single unverified inversion on Chromosome 19 was removed from all further analysis. We also determined whether our identified inversions were present in the GWAS sample itself, a lab population derived from the Quare River high-predation population in 1998 (S29 Fig). Of the 13 inversions segregating in QuH, 11 remained polymorphic in the GWAS fish, sequenced in 2023, indicating that most inversion polymorphisms persisted across roughly 25 years of laboratory culture.

If inversions are associated with local adaptation in relation to upstream-downstream comparisons, we might expect inversions originating prior to the separation of the Trinidad drainages to be associated with replicate high- or low-predation environments across rivers. Alternatively, we would expect more limited distribution for ancient inversions fixed/lost by drift or young inversions arising within specific rivers more recently after colonisation of the island. Although we observe significant genotype-by-environment associations for the inversions on chromosomes 7, 11 and 21, curiously they are not reciprocally fixed, or even substantially more abundant, in all upstream versus downstream populations (S2 Table). Moreover, the remaining inversions present in all three rivers are not associated with upstream or downstream environments (Figs 3 and S30–S32). This suggests for those inversions that are significantly associated with upstream or downstream selection has not fixed them in their favoured environment, possibly due to other environmental factors. For the other inversions observed in all rivers, drift has not eliminated polymorphisms.

**Fig 3:**
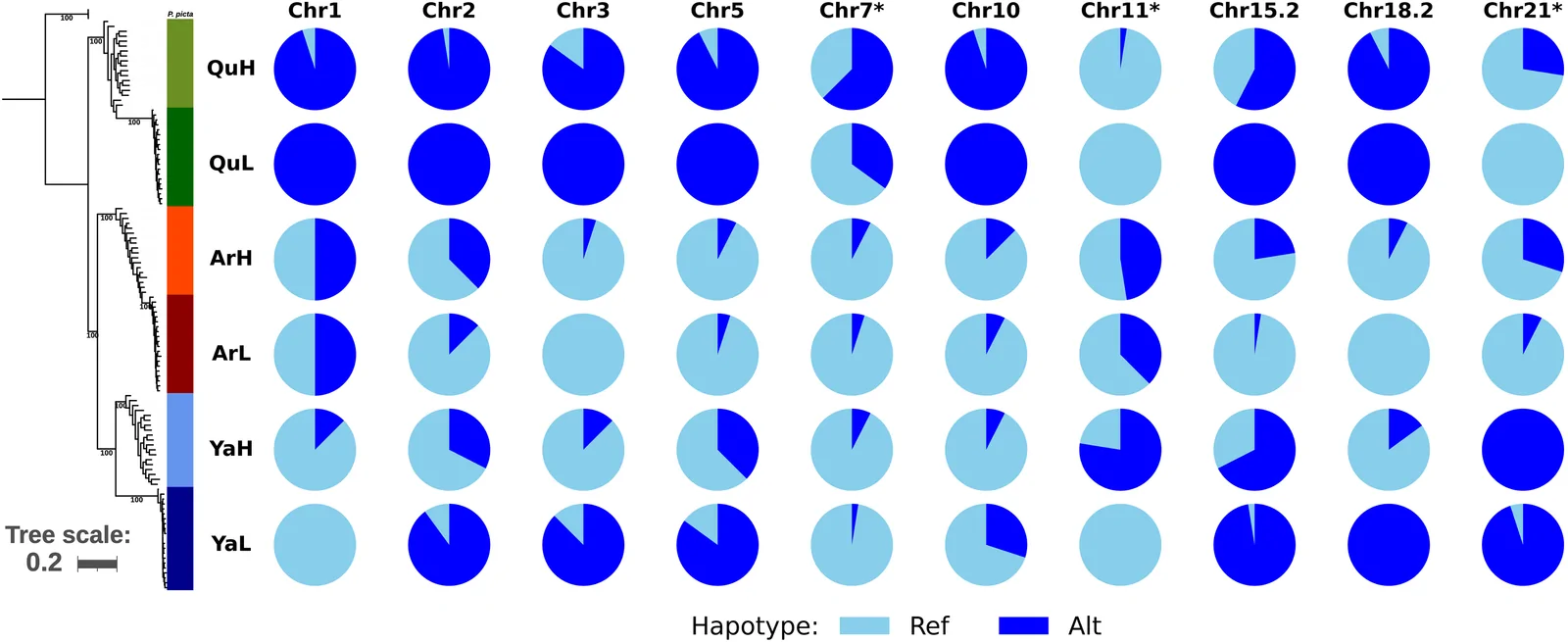
Inversion haplotype diversity in each population. The phylogenetic tree (left) was reconstructed using 270,672 SNPs with RAxML, with bootstrap support of 100 for each population and outgroup nodes. River colours correspond to those used in other figures. For inversions, light blue indicates the reference haplotype, and dark blue indicates the alternative haplotype. Inversions with significant associations with high- and low-predation population environments are shown (*, CMH test, *p* < 0.05, details see S2 Table). The data and code needed to generate this figure can be found at https://doi.org/10.5281/zenodo.22177863.

### Age of inversions

Phylogenetic analysis of our inversions (Figs 2H and panel H in S7–S27 Figs) indicates that inversions are reciprocally monophyletic to the reference in all cases regardless of population sample. This is consistent with an origin of all our inversions before the separation of the Trinidad river drainages. We also dated inversion age following the method in Hill and colleagues [49], and the dates we obtained are also consistent with our phylogenetic analysis (Fig 4A and S3 Table), suggesting they originated before the separation of each watershed we sampled and have been maintained within populations rather than arising in different rivers independently.

**Fig 4:**
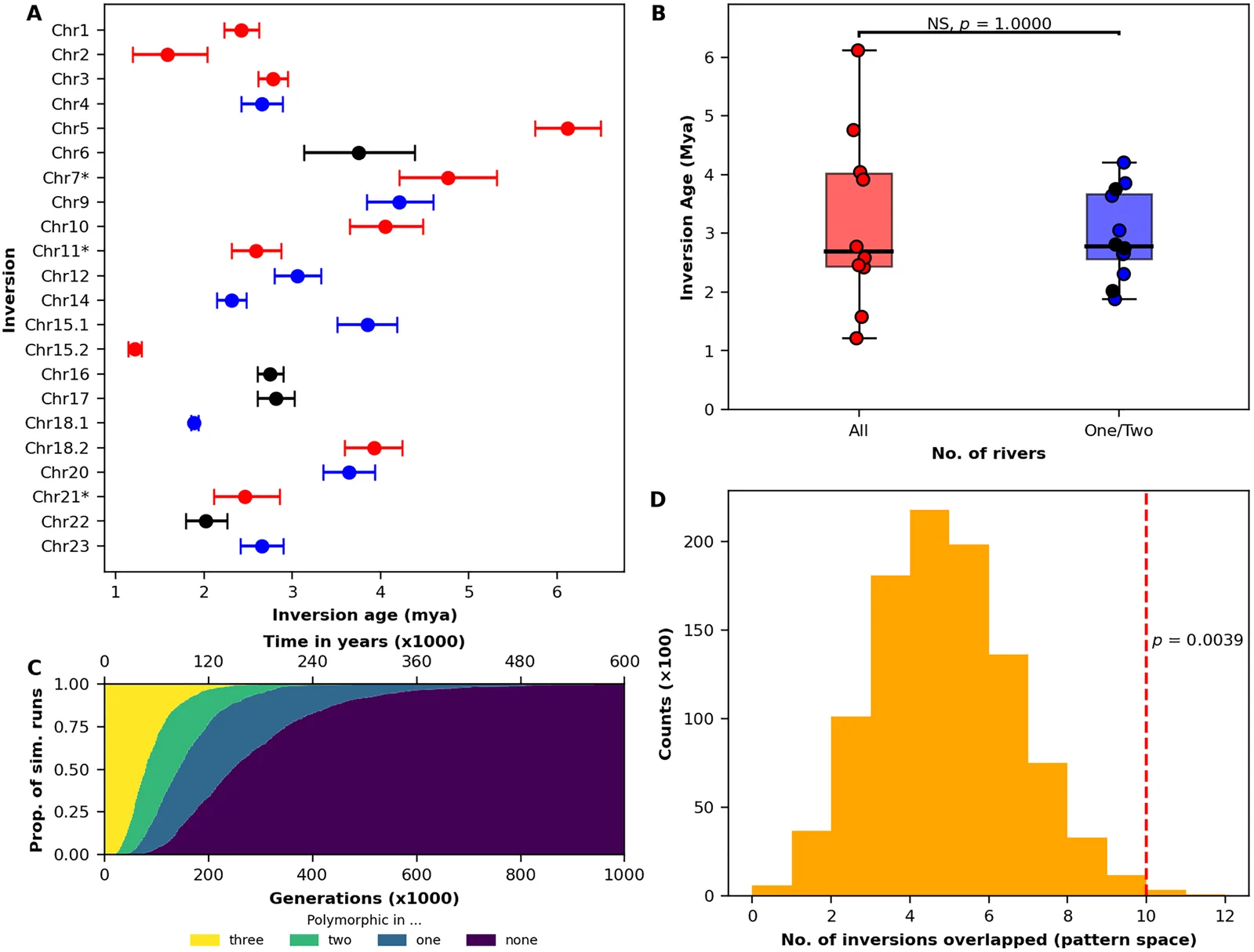
Inversion age and association of colour loci. **A.** Inversion age was calculated following methods described in Hill and colleagues [49], see Materials and methods for details. Nucleotide diversity (*π*) and net divergence (*d*_*xy*_) between two reference and alternate inversion genotypes were calculated in 10 kb windows. Red indicates inversions shared across all three rivers, blue represents inversions shared across two rivers, and black inversions are specific to a single river. Error bars denote 95% confidence intervals. **B.** Boxplots comparing inversion age estimates between inversions shared across all rivers and those shared across one or two rivers. Individual points are coloured to according to inversion sharing category as in A. Horizontal bars indicate statistical comparisons (Mann–Whitney *U* test, NS: not significant, *p* = 1.0). **C.** Forward genetic simulations showing the dynamics of inversion polymorphism across three replicate rivers over 1 million generations (600,000 years) (top x-axis). **D.** Number of inversions overlapped with LD-clumped GWAS loci associated with male ornamentation inferred from Van der Bijl and colleagues [20]. Null distribution was inferred by 100,000 permutation tests. Red dashed line represents the number of observed inversions which capture loci associated with male colour pattern. The data and code needed to generate the plots in this figure can be found at https://doi.org/10.5281/zenodo.22177863.

Although our specific age estimates may be influenced by a host of evolutionary and demographic factors, they are still useful in comparison to each other. Specifically, inversions segregating in one or two rivers are similar in age to those remaining polymorphic in all three (Fig 4B, Mann–Whitney *U* test, *p* = 1.0000), indicating age is not a major predictor of whether an inversion polymorphism is fixed or lost in some rivers. More importantly, for the inversions present in all three rivers, those significantly associated with upstream or downstream environments (on chromosomes 7, 11 and 21) were of similar age (Mann–Whitney *U* test, *p* = 0.8300) to those that were not environmentally associated (Fig 4).

To determine whether our observed polymorphisms are different to that which we would expect under a neutral model, we estimated the likelihood of loss or fixation due to genetic drift (see Materials and methods). Comparing the age of the inversions to forward genetic simulations under neutrality and a conservative demographic scenario suggests that only a small fraction of inversions would remain segregating after 600,000 years, far less than 1 million years (Figs 4C and S33). Under this neutral model, however, nearly all inversions of that age are expected to be polymorphic in at most a single river, as their frequencies drift independently following river divergence. However, the inversions we observed are far more likely to be shared between rivers than restricted to one (18 out of 22 inversions) (Fig 1), a pattern that is highly unlikely to arise under neutrality, suggesting that selection maintains these inversions across rivers and populations.

### Inversions maintained in the population through balancing selection

Guppy females preferentially mate with males with rare colour patterns, resulting in negative frequency-dependent selection for colour patterning [32,33]. Additionally, recent work has suggested that patterning loci are broadly distributed across the guppy genome, spanning the autosomes, X and Y chromosome [20]. To explore whether negative frequency-dependent selection due to sexual selection could maintain inversion variation within populations, we confirmed that our inversions were not Y-derived based on male-to-female read depth ratios (S4 Table). We then examined whether our inversions are more likely to contain loci implicated in male colour pattern variation than we would expect by chance. 68.18% (15 out of 22) of inversions overlap with loci associated with male pattern, as inferred from Van der Bijl and colleagues [20]. Ten inversions overlap with loci associated with pattern space, and 12 inversions overlap with loci associated with the presence/absence of specific ornaments (Figs 2, 4D, and S34A). Conversely, 14.60% (15/103) of pattern space loci fell within inversions, and 10.24% (17/166) of ornament-presence loci. We performed permutation tests separately on aspects of colour, the pattern space (Fig 4D) and the present/absence of individual ornaments (S34A Fig), both of which were statistically significant (permutation test, *p* = 0.0039 and *p* = 0.0080, respectively; Figs 4D and S34A; S5 Table). The pattern loci captured by the inversions in natural populations explain 16.2% of the total phenotypic variation in pattern space in our lab population. This is a third of all variation explained by all pattern space loci, which together explain 47.70% of the phenotypic variation. Similarly, ornament presence loci in inversions explained 13.80% of phenotypic variation, which is 27.00% of the variation of all ornament presence loci (which explain 50.50%). Thus, although inversions harbour a minority of pattern-associated loci, they account for a disproportionate share of whole-pattern variation. Taken together, the identified inversion contain 644 genes in total, which is far less than we would expect by chance based on the genomic distribution (S34B Fig), indicating that our test is conservative and likely an underestimate. Given that there are likely more sexually selected loci in additional populations [57] and that we omitted Chromosome 12 (the sex chromosome) due to its complex structural variation on the Y and between the X and Y and its over-representation of colour loci [20].), these are likely underestimates and sexual selection is likely contributing to the long-term maintenance of inversions in this species.

### Gene content

There are 138 genes in the inversions shared across at least two rivers (S35 Fig). To understand how genes located within the inversions might contribute to differentiation between high- and low-predation populations, we performed a GO term enrichment analysis on inversions (chromosome 7, 11 and 21) that are consistently associated with these populations. The result suggests there is no over-representation of any GO terms. This likely reflects that the biotic and abiotic differences between upstream and downstream environments involve a broad range of traits and biological processes, rather than being driven by a few specific functional pathways.

## Discussion

Using replicate samples of upstream, low predation, and downstream high predation populations across three rivers, the Quare River in the Eastern, Oropouche drainage, the Aripo River in the Western Caroni drainage, and the Yarra River in the Northern Drainage, we identified 22 inversions throughout the guppy genome (Fig 1), of which 10 are maintained in all three sampled rivers. We used linked reads to verify the breakpoints of these inversions, and were able to confirm breakpoints in all but one of them, indicating that they represent inversions (S28 Fig). These are regions with distinct haplotypes consistent with suppressed recombination (Figs 2, 3, and S7–S27), as we also observed elevated *F*_ST_ and LD between our reference and alternate inversions, which also cluster phylogenetically in a pattern consistent with long-term recombination suppression.

High- and low-predation populations experience key biotic (e.g., predation pressure, food availability) and abiotic (e.g., temperature) differences, which affect life-history traits such as body size, male colouration, age at maturity and growth rate [16,24]. Previous studies suggest that guppies from different rivers have undergone convergent phenotypic evolution in response to these differences, however with limited convergence at the genomic level [29–31]. If adaptive and spanning loci related to high- and low-predation syndromes, we might expect inversions to be associated with replicate ecological conditions [58]. We observed significant genotype-by-environment association for three inversions (S2 Table). However, none are reciprocally fixed throughout all three replicate high- and low-predation populations, or even present at high abundance, in our sampled rivers, suggesting that some form of selection prevents fixation of beneficial inversions.

Alternatively, if neutral, we might expect inversions to be fixed or lost in individual rivers due to drift. However, our forward genetic simulations demonstrate that, even under conservative assumptions, it is highly unlikely for a neutral inversion to remain segregating in multiple rivers after 600,000 years (Fig 4C). Despite this prediction, we observed seven inversions that are maintained in all three rivers but show no evidence of genotype-by-environment association, although their estimated ages are comparable to those of inversions exhibiting significant genotype-by-environment associations. Overall, a large proportion of inversions are maintained in all rivers and in most populations (Fig 3). These forward simulations were meant as conservative estimates; while the neutral model accurately captures allele frequency dynamics under drift, it does not account for selection arising from recombination suppression in inversion heterozygotes, including underdominance due to meiotic errors, which would accelerate loss or fixation and make our test more conservative, or associative overdominance arising from the accumulation of recessive deleterious mutations on each haplotype background. The latter could promote the persistence of old, widely distributed inversions maintained as polymorphisms across populations, with no requirement for sexual selection. It is not possible at this point to determine the role of overdominance in causing the patterns we observe, although we would not, under overdominance alone, expect an over-representation of loci associated with male pattern variation in inversions.

The pattern of polymorphism we observed could be due to a recent spread if inversions originated in one river and were trafficked to other watersheds. However, the phylogenetic clustering of our inversions (Figs 2H and panel H in S7–S27 Figs) indicates that the inversions predate the guppy colonisation of different rivers, and our dating of the inversions (Fig 4A) are consistent with this, although the exact dates are likely affected by a range of evolutionary and demographic factors and should be viewed as approximate. After colonisation, inversion genotype frequencies changed over time and accumulated mutations independently in each river. The clear genetic divergence (S1 Fig) among populations suggests there is at best very limited post-divergence gene flow. This pattern is further supported by the inversion phylogeny (Figs 2H, panel H in S7–S27 Figs), in which inversion genotypes from the same population cluster together, albeit sharing the same genotype with populations from other rivers. Such population-specific clustering implies that, despite a shared ancestral origin, inversions evolved independently after establishing in each river in the absence of gene flow, and been maintained by balancing selection.

Our results align with results from previous studies showing that inversion polymorphisms within species can be maintained by balancing selection [5,14,59]. Although other factors may be at play, including complex environmental heterogeneity (e.g., [5], guppies are notable in having a mode of sexual selection whereby male pattern variation is subject to negative frequency-dependent selection [32,33]. Although some previous studies based on the visual assessment of a limited set of male ornaments have emphasised their Y linkage, many other assessments have shown that male colouration also has extensive X and autosomal components [57,60–64,] and a recent agnostic study identified a highly polygenic structure spanning the genome for male pattern variation [20]. We therefore assessed the overlap between autosomal inversions and male sexually-selected pattern loci potentially subject to negative frequency-dependent selection from Van der Bijl and colleagues [20]. Although identification of male pattern loci was limited to one population, and broader analyses of additional populations will likely yield additional sites, we observe a significant association of inversions with both the overall pattern space as well as the presence/absence of specific ornaments (Figs 4D and S34A). Given that there are likely more sexually selected loci in additional populations [57], and that we omitted Chromosome 12 (the sex chromosome) due to its complex structural variation on the Y and between the X and Y and its over-representation of colour loci [20,65], these are likely underestimates. However, our estimates may be anti-conservative in that we are unable to control for recombination-rate variation.

Our results suggest that a large proportion of inversions in the guppy genome may have captured sexually selected loci (Fig 1) that are subject to negative frequency-dependent selection [32,33], where rare phenotypes are more selected by mates. It is not possible to differentiate whether the inversions formed to capture existing loci involved in sexual selection, or whether existing inversions captured loci once formed. Regardless, our results suggest ancient inversions can exhibit variable evolutionary trajectories across populations, reflecting a dynamic balance between structural genomic constraints and sexual selection pressure. Thus, some inversions may be maintained by negative frequency-dependent sexual selection, and this dynamic thereby facilitates the long-term maintenance of multiple inversion genotypes within and across populations, highlighting the role of sexual selection not only in structural genomic variation but also in the maintenance of genetic diversity.

Overall, our results highlight how inversions underlying ecologically and sexually important traits persist, potentially contributing to remarkable phenotypic and genomic diversity observed in natural populations. Sexual selection is a major force shaping animal genomes, and the negative frequency-dependent selection seen in guppies is a characteristic of many other sexual selection systems, including *Drosophila* [66] and side-blotched lizards [67], to name just a few. It is likely that mate preference for rarity or novelty is far more widespread than currently realised, as the relevant polymorphism important in mate choice in a given animal species is often not easily detected by human senses [68]. Given this, our results suggest that sexual selection may play a broader role in maintaining inversions in many other sexually selected species.

### Ethics statement

Animal care protocols were approved by the University of British Columbia animal care committee (A22-0239), and permits for sampling fish were obtained from Trinidad Ministry of Agriculture, Land and Fisheries and Indar Ramnarine.

## Supporting information

S1 Table Barcode enrichment summary statistics and physical location of inversion breakpoints. (DOCX)

S2 Table Genotype-by-environment test on convergent haplotype differing between high- and low-predation populations. (DOCX)

S3 Table Estimated age of inversions in millions of years. (DOCX)

S4 Table Male-to-female read depth ratio of inversions. (DOCX)

S5 Table Number of LD-clumped hits within each inversion. (DOCX)

S1 Fig Population structure of samples. PCA of genome **(A)** and sex chromosome (chromosome 12) **(B)**. ArH/ArL: Aripo high- and low-predation populations; QuH/QuL: Quare high- and low-predation populations; YaH/YaL: Yarra high- and low-predation populations. **(C)** Admixture result further supports the distinct structure of samples from each river. The data and code needed to generate this figure can be found at https://doi.org/10.5281/zenodo.22177863.
(TIF)

S2 Fig Genetic differentiation between high- and low-predation populations in three rivers. Blue blocks indicate the putative SV regions. Dashed line represents the average *F*_ST_ across genome. The data and code needed to generate this figure can be found at https://doi.org/10.5281/zenodo.22177863.
(TIF)

S3 Fig Nucleotide diversity of guppies in three rivers. Blue blocks on the top of each panel indicate the putative inverted regions. Dashed line is the average across the genomic regions. The data and code needed to generate this figure can be found at https://doi.org/10.5281/zenodo.22177863.
(TIF)

S4 Fig Demographic history of Trinidadian guppies. PSMC results were visualised with **0.6 (210 days)** year as generation time and mutation rate 1.35 * 10^−08^/site/generation. This trend of demographic history is similar across different populations. High-predation populations (ArH, QuH, and YaH) show higher effective population size than their low-predation counterparts (ArL, QuL and YaL). The data and code needed to generate this figure can be found at https://doi.org/10.5281/zenodo.22177863.
(TIF)

S5 Fig LD decay across all populations. The plot shows the relationship between mean *r*^2^ and physical distance (kb) for six populations (ArH, ArL, QuH, QuL, YaH, and YaL). LD decreases with increasing distance between SNPs, with high-predation populations (ArH, QuH, and YaH) showing lower LD than their low-predation counterparts (ArL, QuL and YaL). These distinct decay patterns reflect variation in recombination rate and demographic history. The data and code needed to generate this figure can be found at https://doi.org/10.5281/zenodo.22177863.
(TIF)

S6 Fig Phylogeny of 22 identified inversions. Each panel shows a phylogenetic tree of an inversion reconstructed using IQ-TREE3. Two trees of each inversion inferred, one under the GTR model (left) and the other using ModelFinder’s automated model selection (AUTO) (right), are quantitatively similar. The data and code needed to generate this figure can be found at https://doi.org/10.5281/zenodo.22177863.
(TIF)

S7 Fig Chromosome 1, 10.33–11.61 Mb (Chr1). **A.** MDS01 along chromosome 3 with inversion highlighted in blue. **B.** PCA of the inversion shows three distinct clusters, representing the reference homozygote, heterozygote, and alternative homozygote genotypes. Inset PCA panel, which is restricted to individuals classified as alternative homozygotes (orange), shows the presence of nested sub-inversions within the reference haplotype. **C.** *F*_ST_ between two homozygous inversion genotypes (cluster 0 and cluster 2). **D.** Average proportion of heterozygous SNPs across individuals in three clusters corresponding to panel B. **E.** LD plot. **F.** Proportion of three different inversion genotypes in high- and low-predation populations with inversion genotypes shown. NS: not significant deviated from HWE. **G.** Genotype plot of all individuals in the focal population. Grey, homozygous reference alleles (0/0); orange, heterozygous alleles (0/1); green, homozygous alternative alleles (1/1); white, missing genotype (./.). *X* axis, SNPs; *Y* axis, sample names; Population colour bar, population colour corresponds to these in Fig 1; Cluster colour bar, corresponds to those in panel B. **H.** Phylogeny of inversions including individuals from cluster 0 and cluster 2. Cluster colour and population colour are the same as those in Fig 2G. The data and code needed to generate this figure can be found at https://doi.org/10.5281/zenodo.22177863.
(TIF)

S8 Fig Chromosome 2, 10.79–11.09 Mb (Chr2). **A.** MDS01 along chromosome 2 with inversion highlighted in blue. **B.** PCA of the inversion shows three distinct clusters, representing the reference homozygote, heterozygote, and alternative homozygote genotypes. **C.** *F*_ST_ between two homozygous inversion genotypes (cluster 0 and cluster 2). **D.** Average proportion of heterozygous SNPs across individuals in three clusters corresponding to panel B. **E.** LD plot. **F.** Proportion of three different inversion genotypes in high- and low-predation populations. NS: not significant deviated from HWE. **G.** Genotype plot of all individuals in the focal population. Grey, homozygous reference alleles (0/0); orange, heterozygous alleles (0/1); green, homozygous alternative alleles (1/1); white, missing genotype (./.). *X* axis, SNPs; *Y* axis, sample names; Population colour bar, population colour corresponds to these in Fig 1; Cluster colour bar, corresponds to those in panel B. **H.** Phylogeny of inversions including individuals from cluster 0 and cluster 2. Cluster colour and population colour are the same as those in Fig 2G. The data and code needed to generate this figure can be found at https://doi.org/10.5281/zenodo.22177863.
(TIF)

S9 Fig Chromosome 3, 13.27–14.86 Mb (Chr3). **A.** MDS01 along chromosome 3 with inversion highlighted in blue. **B.** PCA of the inversion shows three distinct clusters, representing the reference homozygote, heterozygote, and alternative homozygote genotypes. **C.** *F*_ST_ between two homozygous inversion genotypes (cluster 0 and cluster 2). **D.** Average proportion of heterozygous SNPs across individuals in three clusters corresponding to panel B. **E.** LD plot. **F.** Proportion of three different inversion genotypes in high- and low-predation populations. NS: not significant deviated from HWE. **G.** Genotype plot of all individuals in the focal population. Grey, homozygous reference alleles (0/0); orange, heterozygous alleles (0/1); green, homozygous alternative alleles (1/1); white, missing genotype (./.). *X* axis, SNPs; *Y* axis, sample names; Population colour bar, population colour corresponds to these in Fig 1; Cluster colour bar, corresponds to those in panel B. **H.** Phylogeny of inversions including individuals from cluster 0 and cluster 2. Cluster colour and population colour are the same as those in Fig 2G. The data and code needed to generate this figure can be found at https://doi.org/10.5281/zenodo.22177863.
(TIF)

S10 Fig Chromosome 4, 29.95–31.08 Mb (Chr4). **A.** MDS01 along chromosome 4 with inversion highlighted in blue. **B.** PCA of the inversion shows three distinct clusters, representing the reference homozygote, heterozygote, and alternative homozygote genotypes. **C.** *F*_ST_ between two homozygous inversion genotypes (cluster 0 and cluster 2). **D.** Average proportion of heterozygous SNPs across individuals in three clusters corresponding to panel B. E. LD plot. **F.** Proportion of three different inversion genotypes in high- and low-predation populations. NS: not significant deviated from HWE. **G.** Genotype plot of all individuals in the focal population. Grey, homozygous reference alleles (0/0); orange, heterozygous alleles (0/1); green, homozygous alternative alleles (1/1); white, missing genotype (./.). *X* axis, SNPs; *Y* axis, sample names; Population colour bar, population colour corresponds to these in Fig 1; Cluster colour bar, corresponds to those in panel B. **H.** Phylogeny of inversions including individuals from cluster 0 and cluster 2. Cluster colour and population colour are the same as those in Fig 2G. The data and code needed to generate this figure can be found at https://doi.org/10.5281/zenodo.22177863.
(TIF)

S11 Fig Chromosome 6, 5.11–5.30 Mb (Chr6). **A.** MDS01 along chromosome 6 with inversion highlighted in blue. **B.** PCA of the inversion shows three distinct clusters, representing the reference homozygote, heterozygote, and alternative homozygote genotypes. **C.** *F*_ST_ between two homozygous inversion genotypes (cluster 0 and cluster 2). **D.** Average proportion of heterozygous SNPs across individuals in three clusters corresponding to panel B. **E.** LD plot. **F.** Proportion of three different inversion genotypes in high- and low-predation populations. NS: not significant deviated from HWE. **G.** Genotype plot of all individuals in the focal population. Grey, homozygous reference alleles (0/0); orange, heterozygous alleles (0/1); green, homozygous alternative alleles (1/1); white, missing genotype (./.). *X* axis, SNPs; *Y* axis, sample names; Population colour bar, population colour corresponds to these in Fig 1; Cluster colour bar, corresponds to those in panel B. **H.** Phylogeny of inversions including individuals from cluster 0 and cluster 2. Cluster colour and population colour are the same as those in Fig 2G. The data and code needed to generate this figure can be found at https://doi.org/10.5281/zenodo.22177863.
(TIF)

S12 Fig Chromosome 7, 6.98–7.51 Mb (Chr7). **A.** MDS01 along chromosome 7 with inversion highlighted in blue. **B.** PCA of the inversion shows three distinct clusters, representing the reference homozygote, heterozygote, and alternative homozygote genotypes. **C.** *F*_ST_ between two homozygous inversion genotypes (cluster 0 and cluster 2). **D.** Average proportion of heterozygous SNPs across individuals in three clusters corresponding to panel B. **E.** LD plot. **F.** Proportion of three different inversion genotypes in high- and low-predation populations. NS: not significant deviated from HWE. **G.** Genotype plot of all individuals in the focal population. Grey, homozygous reference alleles (0/0); orange, heterozygous alleles (0/1); green, homozygous alternative alleles (1/1); white, missing genotype (./.). *X* axis, SNPs; *Y* axis, sample names; Population colour bar, population colour corresponds to these in Fig 1; Cluster colour bar, corresponds to those in panel B. **H.** Phylogeny of inversions including individuals from cluster 0 and cluster 2. Cluster colour and population colour are the same as those in Fig 2G. The data and code needed to generate this figure can be found at https://doi.org/10.5281/zenodo.22177863.
(TIF)

S13 Fig Chromosome 9, 30.20–31.01 Mb (Chr9). **A.** MDS01 along chromosome 9 with inversion highlighted in blue. **B.** PCA of the inversion shows three distinct clusters, representing the reference homozygote, heterozygote, and alternative homozygote genotypes. **C.** *F*_ST_ between two homozygous inversion genotypes (cluster 0 and cluster 2). **D.** Average proportion of heterozygous SNPs across individuals in three clusters corresponding to panel B. **E.** LD plot. **F.** Proportion of three different inversion genotypes in high- and low-predation populations. NS: not significant deviated from HWE. **G.** Genotype plot of all individuals in the focal population. Grey, homozygous reference alleles (0/0); orange, heterozygous alleles (0/1); green, homozygous alternative alleles (1/1); white, missing genotype (./.). *X* axis, SNPs; *Y* axis, sample names; Population colour bar, population colour corresponds to these in Fig 1; Cluster colour bar, corresponds to those in panel B. H. Phylogeny of inversions including individuals from cluster 0 and cluster 2. Cluster colour and population colour are the same as those in Fig 2G. The data and code needed to generate this figure can be found at https://doi.org/10.5281/zenodo.22177863.
(TIF)

S14 Fig Chromosome 10, 8.64–9.18 Mb (Chr10). **A.** MDS01 along chromosome 10 with inversion highlighted in blue. **B.** PCA of the inversion shows three distinct clusters, representing the reference homozygote, heterozygote, and alternative homozygote genotypes. **C.** *F*_ST_ between two homozygous inversion genotypes (cluster 0 and cluster 2). **D.** Average proportion of heterozygous SNPs across individuals in three clusters corresponding to panel B. **E.** LD plot. **F.** Proportion of three different inversion genotypes in high- and low-predation populations. NS: not significant deviated from HWE. **G.** Genotype plot of all individuals in the focal population. Grey, homozygous reference alleles (0/0); orange, heterozygous alleles (0/1); green, homozygous alternative alleles (1/1); white, missing genotype (./.). *X* axis, SNPs; *Y* axis, sample names; Population colour bar, population colour corresponds to these in Fig 1; Cluster colour bar, corresponds to those in panel B. **H.** Phylogeny of inversions including individuals from cluster 0 and cluster 2. Cluster colour and population colour are the same as those in Fig 2G. The data and code needed to generate this figure can be found at https://doi.org/10.5281/zenodo.22177863.
(TIF)

S15 Fig Chromosome 11, 7.22–7.50 Mb (Chr11). **A.** MDS01 along chromosome 11 with inversion highlighted in blue. **B.** PCA of the inversion shows three distinct clusters, representing the reference homozygote, heterozygote, and alternative homozygote genotypes. **C.** *F*_ST_ between two homozygous inversion genotypes (cluster 0 and cluster 2). **D.** Average proportion of heterozygous SNPs across individuals in three clusters corresponding to panel B. **E.** LD plot. **F.** Proportion of three different inversion genotypes in high- and low-predation populations. NS: not significant deviated from HWE. **G.** Genotype plot of all individuals in the focal population. Grey, homozygous reference alleles (0/0); orange, heterozygous alleles (0/1); green, homozygous alternative alleles (1/1); white, missing genotype (./.). *X* axis, SNPs; *Y* axis, sample names; Population colour bar, population colour corresponds to these in Fig 1; Cluster colour bar, corresponds to those in panel B. **H.** Phylogeny of inversions including individuals from cluster 0 and cluster 2. Cluster colour and population colour are the same as those in Fig 2G. The data and code needed to generate this figure can be found at https://doi.org/10.5281/zenodo.22177863.
(TIF)

S16 Fig Chromosome 12, 10.37–10.96 Mb (Chr12). **A.** MDS01 along chromosome 12 with inversion highlighted in blue. **B.** PCA of the inversion shows three distinct clusters, representing the reference homozygote, heterozygote, and alternative homozygote genotypes. **C.** *F*_ST_ between two homozygous inversion genotypes (cluster 0 and cluster 2). **D.** Average proportion of heterozygous SNPs across individuals in three clusters corresponding to panel B. **E.** LD plot. **F.** Proportion of three different inversion genotypes in high- and low-predation populations. NS: not significant deviated from HWE. **G.** Genotype plot of all individuals in the focal population. Grey, homozygous reference alleles (0/0); orange, heterozygous alleles (0/1); green, homozygous alternative alleles (1/1); white, missing genotype (./.). *X* axis, SNPs; *Y* axis, sample names; Population colour bar, population colour corresponds to these in Fig 1; Cluster colour bar, corresponds to those in panel B. **H.** Phylogeny of inversions including individuals from cluster 0 and cluster 2. Cluster colour and population colour are the same as those in Fig 2G. The data and code needed to generate this figure can be found at https://doi.org/10.5281/zenodo.22177863.
(TIF)

S17 Fig Chromosome 14, 26.81–28.03 Mb (Chr14). **A.** MDS01 along chromosome 14 with inversion highlighted in blue. **B.** PCA of the inversion shows three distinct clusters, representing the reference homozygote, heterozygote, and alternative homozygote genotypes. **C.** *F*_ST_ between two homozygous inversion genotypes (cluster 0 and cluster 2). **D.** Average proportion of heterozygous SNPs across individuals in three clusters corresponding to panel B. **E.** LD plot. **F.** Proportion of three different inversion genotypes in high- and low-predation populations. NS: not significant deviated from HWE. **G.** Genotype plot of all individuals in the focal population. Grey, homozygous reference alleles (0/0); orange, heterozygous alleles (0/1); green, homozygous alternative alleles (1/1); white, missing genotype (./.). *X* axis, SNPs; *Y* axis, sample names; Population colour bar, population colour corresponds to these in Fig 1; Cluster colour bar, corresponds to those in panel B. **H.** Phylogeny of inversions including individuals from cluster 0 and cluster 2. Cluster colour and population colour are the same as those in Fig 2G. The data and code needed to generate this figure can be found at https://doi.org/10.5281/zenodo.22177863.
(TIF)

S18 Fig Chromosome 15, 4.66–5.14 Mb (Chr15.1). **A.** MDS01 along chromosome 15 with inversion highlighted in blue. **B.** PCA of the inversion shows three distinct clusters, representing the reference homozygote, heterozygote, and alternative homozygote genotypes. **C.** *F*_ST_ between two homozygous inversion genotypes (cluster 0 and cluster 2). **D.** Average proportion of heterozygous SNPs across individuals in three clusters corresponding to panel B. E. LD plot. **F.** Proportion of three different inversion genotypes in high- and low-predation populations. NS: not significant deviated from HWE. **G.** Genotype plot of all individuals in the focal population. Grey, homozygous reference alleles (0/0); orange, heterozygous alleles (0/1); green, homozygous alternative alleles (1/1); white, missing genotype (./.). *X* axis, SNPs; *Y* axis, sample names; Population colour bar, population colour corresponds to these in Fig 1; Cluster colour bar, corresponds to those in panel B. **H.** Phylogeny of inversions including individuals from cluster 0 and cluster 2. Cluster colour and population colour are the same as those in Fig 2G. The data and code needed to generate this figure can be found at https://doi.org/10.5281/zenodo.22177863.
(TIF)

S19 Fig Chromosome 15, 7.55–8.71 Mb (Chr15.2). **A.** MDS01 along chromosome 15 with inversion highlighted in blue. **B.** PCA of the inversion shows three distinct clusters, representing the reference homozygote, heterozygote, and alternative homozygote genotypes. **C.** *F*_ST_ between two homozygous inversion genotypes (cluster 0 and cluster 2). **D.** Average proportion of heterozygous SNPs across individuals in three clusters corresponding to panel B. **E.** LD plot. **F.** Proportion of three different inversion genotypes in high- and low-predation populations. NS: not significant deviated from HWE. G. Genotype plot of all individuals in the focal population. Grey, homozygous reference alleles (0/0); orange, heterozygous alleles (0/1); green, homozygous alternative alleles (1/1); white, missing genotype (./.). *X* axis, SNPs; *Y* axis, sample names; Population colour bar, population colour corresponds to these in Fig 1; Cluster colour bar, corresponds to those in panel B. H. Phylogeny of inversions including individuals from cluster 0 and cluster 2. Cluster colour and population colour are the same as those in Fig 2G. The data and code needed to generate this figure can be found at https://doi.org/10.5281/zenodo.22177863.
(TIF)

S20 Fig Chromosome 16, 29.77–31.49 Mb (Chr16). **A.** MDS01 along chromosome 16 with inversion highlighted in blue. **B.** PCA of the inversion shows three distinct clusters, representing the reference homozygote, heterozygote, and alternative homozygote genotypes. **C.** *F*_ST_ between two homozygous inversion genotypes (cluster 0 and cluster 2). **D.** Average proportion of heterozygous SNPs across individuals in three clusters corresponding to panel B. **E.** LD plot. **F.** Proportion of three different inversion genotypes in high- and low-predation populations. NS: not significant deviated from HWE. **G.** Genotype plot of all individuals in the focal population. Grey, homozygous reference alleles (0/0); orange, heterozygous alleles (0/1); green, homozygous alternative alleles (1/1); white, missing genotype (./.). *X* axis, SNPs; *Y* axis, sample names; Population colour bar, population colour corresponds to these in Fig 1; Cluster colour bar, corresponds to those in panel B. **H.** Phylogeny of inversions including individuals from cluster 0 and cluster 2. Cluster colour and population colour are the same as those in Fig 2G. The data and code needed to generate this figure can be found at https://doi.org/10.5281/zenodo.22177863.
(TIF)

S21 Fig Chromosome 17, 9.48–10.43 Mb (Chr17). **A.** MDS01 along chromosome 17 with inversion highlighted in blue. **B.** PCA of the inversion shows three distinct clusters, representing the reference homozygote, heterozygote, and alternative homozygote genotypes. **C.** *F*_ST_ between two homozygous inversion genotypes (cluster 0 and cluster 2). **D.** Average proportion of heterozygous SNPs across individuals in three clusters corresponding to panel B. **E.** LD plot. **F.** Proportion of three different inversion genotypes in high- and low-predation populations. NS: not significant deviated from HWE. **G.** Genotype plot of all individuals in the focal population. Grey, homozygous reference alleles (0/0); orange, heterozygous alleles (0/1); green, homozygous alternative alleles (1/1); white, missing genotype (./.). *X* axis, SNPs; *Y* axis, sample names; Population colour bar, population colour corresponds to these in Fig 1; Cluster colour bar, corresponds to those in panel B. **H.** Phylogeny of inversions including individuals from cluster 0 and cluster 2. Cluster colour and population colour are the same as those in Fig 2G. The data and code needed to generate this figure can be found at https://doi.org/10.5281/zenodo.22177863.
(TIF)

S22 Fig Chromosome 18, 0.08–0.38 Mb (Chr18.1). **A.** MDS01 along chromosome 18 with inversion highlighted in blue. **B.** PCA of the inversion shows three distinct clusters, representing the reference homozygote, heterozygote, and alternative homozygote genotypes. **C.** *F*_ST_ between two homozygous inversion genotypes (cluster 0 and cluster 2). **D.** Average proportion of heterozygous SNPs across individuals in three clusters corresponding to panel B. **E.** LD plot. **F.** Proportion of three different inversion genotypes in high- and low-predation populations. NS: not significant deviated from HWE. **G.** Genotype plot of all individuals in the focal population. Grey, homozygous reference alleles (0/0); orange, heterozygous alleles (0/1); green, homozygous alternative alleles (1/1); white, missing genotype (./.). *X* axis, SNPs; *Y* axis, sample names; Population colour bar, population colour corresponds to these in Fig 1; Cluster colour bar, corresponds to those in panel B. **H.** Phylogeny of inversions including individuals from cluster 0 and cluster 2. Cluster colour and population colour are the same as those in Fig 2G. The data and code needed to generate this figure can be found at https://doi.org/10.5281/zenodo.22177863.
(TIF)

S23 Fig Chromosome 18, 0.51–1.11 Mb (Chr18.2). **A.** MDS01 along chromosome 18 with inversion highlighted in blue. **B.** PCA of the inversion shows three distinct clusters, representing the reference homozygote, heterozygote, and alternative homozygote genotypes. **C.** *F*_ST_ between two homozygous inversion genotypes (cluster 0 and cluster 2). **D.** Average proportion of heterozygous SNPs across individuals in three clusters corresponding to panel B. **E.** LD plot. **F.** Proportion of three different inversion genotypes in high- and low-predation populations. NS: not significant deviated from HWE. **G.** Genotype plot of all individuals in the focal population. Grey, homozygous reference alleles (0/0); orange, heterozygous alleles (0/1); green, homozygous alternative alleles (1/1); white, missing genotype (./.). *X* axis, SNPs; *Y* axis, sample names; Population colour bar, population colour corresponds to these in Fig 1; Cluster colour bar, corresponds to those in panel B. **H.** Phylogeny of inversions including individuals from cluster 0 and cluster 2. Cluster colour and population colour are the same as those in Fig 2G. The data and code needed to generate this figure can be found at https://doi.org/10.5281/zenodo.22177863.
(TIF)

S24 Fig Chromosome 20, 0.38–1.12 Mb (Chr20). **A.** MDS01 along chromosome 20 with inversion highlighted in blue. **B.** PCA of the inversion shows three distinct clusters, representing the reference homozygote, heterozygote, and alternative homozygote genotypes. **C.** *F*_ST_ between two homozygous inversion genotypes (cluster 0 and cluster 2). **D.** Average proportion of heterozygous SNPs across individuals in three clusters corresponding to panel B. **E.** LD plot. **F.** Proportion of three different inversion genotypes in high- and low-predation populations. NS: not significant deviated from HWE. **G.** Genotype plot of all individuals in the focal population. Grey, homozygous reference alleles (0/0); orange, heterozygous alleles (0/1); green, homozygous alternative alleles (1/1); white, missing genotype (./.). *X* axis, SNPs; *Y* axis, sample names; Population colour bar, population colour corresponds to these in Fig 1; Cluster colour bar, corresponds to those in panel B. **H.** Phylogeny of inversions including individuals from cluster 0 and cluster 2. Cluster colour and population colour are the same as those in Fig 2G. The data and code needed to generate the plots in this figure can be found at https://doi.org/10.5281/zenodo.22177863.
(TIF)

S25 Fig Chromosome 21, 24.15–24.75 Mb (Chr21). **A.** MDS01 along Chromosome 21 with inversion highlighted in blue. **B.** PCA of the inversion shows three distinct clusters, representing the reference homozygote, heterozygote, and alternative homozygote genotypes. **C.** *F*_ST_ between two homozygous inversion genotypes (cluster 0 and cluster 2). **D.** Average proportion of heterozygous SNPs across individuals in three clusters corresponding to panel B. **E.** LD plot. **F.** Proportion of three different inversion genotypes in high- and low-predation populations. NS: not significant deviated from HWE. **G.** Genotype plot of all individuals in the focal population. Grey, homozygous reference alleles (0/0); orange, heterozygous alleles (0/1); green, homozygous alternative alleles (1/1); white, missing genotype (./.). *X* axis, SNPs; *Y* axis, sample names; Population colour bar, population colour corresponds to these in Fig 1; Cluster colour bar, corresponds to those in panel B. **H.** Phylogeny of inversions including individuals from cluster 0 and cluster 2. Cluster colour and population colour are the same as those in Fig 2G. The data and code needed to generate this figure can be found at https://doi.org/10.5281/zenodo.22177863.
(TIF)

S26 Fig Chromosome 22, 0.01–1.48 Mb. A. MDS01 along chromosome 22 with inversion highlighted in blue. **B.** PCA of the inversion shows three distinct clusters, representing the reference homozygote, heterozygote, and alternative homozygote genotypes. **C.** *F*_ST_ between two homozygous inversion genotypes (cluster 0 and cluster 2). **D.** Average proportion of heterozygous SNPs across individuals in three clusters corresponding to panel B. **E.** LD plot. **F.** Proportion of three different inversion genotypes in high- and low-predation populations. NS: not significant deviated from HWE. **G.** Genotype plot of all individuals in the focal population. Grey, homozygous reference alleles (0/0); orange, heterozygous alleles (0/1); green, homozygous alternative alleles (1/1); white, missing genotype (./.). *X* axis, SNPs; *Y* axis, sample names; Population colour bar, population colour corresponds to these in Fig 1; Cluster colour bar, corresponds to those in panel B. **H.** Phylogeny of inversions including individuals from cluster 0 and cluster 2. Cluster colour and population colour are the same as those in Fig 2G. The data and code needed to generate this figure can be found at https://doi.org/10.5281/zenodo.22177863.
(TIF)

S27 Fig Chromosome 23, 12.49–13.20 Mb. A. MDS01 along chromosome 23 with inversion highlighted in blue. **B.** PCA of the inversion shows three distinct clusters, representing the reference homozygote, heterozygote, and alternative homozygote genotypes. **C.** *F*_ST_ between two homozygous inversion genotypes (cluster 0 and cluster 2). **D.** Average proportion of heterozygous SNPs across individuals in three clusters corresponding to panel B. **E.** LD plot. **F.** Proportion of three different inversion genotypes in high- and low-predation populations. NS: not significant deviated from HWE. **G.** Genotype plot of all individuals in the focal population. Grey, homozygous reference alleles (0/0); orange, heterozygous alleles (0/1); green, homozygous alternative alleles (1/1); white, missing genotype (./.). *X* axis, SNPs; *Y* axis, sample names; Population colour bar, population colour corresponds to these in Fig 1; Cluster colour bar, corresponds to those in panel B. **H.** Phylogeny of inversions including individuals from cluster 0 and cluster 2. Cluster colour and population colour are the same as those in Fig 2G. The data and code needed to generate this figure can be found at https://doi.org/10.5281/zenodo.22177863.
(TIF)

S28 Fig Breakpoints of 22 identified inversions (A–V). Arrows in each subplot indicate the breakpoint locations for the corresponding inversion. Numbers of barcodes sharing across genomic positions were obtained using LongRanger and Loupe v2.12.2. The data and code needed to generate this figure can be found at https://doi.org/10.5281/zenodo.22177863.
(TIF)

S29 Fig Evidence for the presence of inversions in the QuH-derived lab population used in Van der Bijl and colleagues [20]. All inversions except 18.1 and 18.2 show evidence for two haplotypes along PC1 with an additional cluster for heterozygotes. Left panels show individual males in PCA coordinates space, with colour and shape showing cluster membership. Right panels display their heterozygosity as boxplots. Boxplots show the median and quartiles as the box, whiskers extend to the furthest observation within 1.5x the interquartile range, and points outside the whiskers are shown individually. The data and code needed to generate this figure can be found at https://doi.org/10.5281/zenodo.22177863.
(TIF)

S30 Fig Haplotype frequency of polymorphic inversions across one and two rivers. Each row represents a population, with its names corresponding to the river names. The data and code needed to generate this figure can be found at https://doi.org/10.5281/zenodo.22177863.
(TIF)

S31 Fig Genotype frequency of polymorphic inversions across three rivers. Each row represents a population, with its names corresponding to the river names. * shows the significant different between high- and low-predation populations across rivers (details see S2 Table and Fig 3). The data and code needed to generate this figure can be found at https://doi.org/10.5281/zenodo.22177863.
(TIF)

S32 Fig Genotype frequency of inversions in both one and two rivers. Each row represents a population, with its names corresponding to the river names. The data and code needed to generate this figure can be found at https://doi.org/10.5281/zenodo.22177863.
(TIF)

S33 Fig Forward simulation showing dynamics of inversions in three independent rivers under neutrality in 1 million generations, equivalent to 600,000 years, tested with scaling factor *k =* 20 (top), *k =* 10 (median) and *k =* 5 (bottom). The data and code needed to generate this figure can be found at https://doi.org/10.5281/zenodo.22177863.
(TIF)

S34 Fig Null distribution of SNP–inversion (A) and gene-inversion (B) overlaps from 100,000 permutation tests. Permutation test was conducted on LD-clumped GWAS SNPs associated with present/absent of colour ornaments. Red dashed line represents the number of observed inversions which capture colour loci associated with present/absent of colour ornaments (A), and the number of observed genes in inversion regions (B). The data and code needed to generate this figure can be found at https://doi.org/10.5281/zenodo.22177863.
(TIF)

S35 Fig Upset plot shows the number of genes in inversions from each river and shared across rivers. In total, there are 138 genes found in inversion regions shared across three rivers. The data and code needed to generate this figure can be found at https://doi.org/10.5281/zenodo.22177863.
(TIF)

## Abbreviations

CMH: Cochran–Mantel–Haenszel
CV: cross-validation
HMM: Hidden Markov Model
LD: linkage disequilibrium
PCs: principal components
