# Correction: Ancient inversion polymorphisms associate with sexually selected traits across natural guppy populations

**DOI:** 10.1371/journal.pbio.3004110

**Published:** 2026-11-13

**Authors:** The PLOS Biology Staff

## Notice of Republication

This article was republished on November 3rd, 2026, to include the missing Supplemental Tables. The publisher apologizes for the errors. Please download this article again to view the correct version. The originally published, uncorrected article and the republished, corrected articles are provided here for reference.

## Supporting information

S1 File Originally published, uncorrected article. (PDF)

S2 File Republished, corrected article. (PDF)
