# Supplementary material for: Ancient inversion polymorphisms associate with sexually selected traits across natural guppy populations
Source: PLoS Biol. 2026 Sep 23;24(9):e3004024. doi: 10.1371/journal.pbio.3004024 (PMC13626464; doi:10.1371/journal.pbio.3004024)
Supplement: Supplementary file 1 [file pbio.3004024.s036.docx]

**S1 Table. Barcode enrichment summary statistics and physical location of inversion breakpoints.**

| Regions1 | Start_  barcode | End_  barcode | Start_ localPCA | End_  localPCA | Mean* | Mean_  nonzeros | Std* | # of windows_  nonzero | # of windows_  total | Nonzero  ratio (%) |
| --- | --- | --- | --- | --- | --- | --- | --- | --- | --- | --- |
| Chr1_Inv | 10228968 | 11655288 | 10328090 | 11605890 | 0.5626 | 3.6495 | 3.2260 | 20539 | 133225 | 15.4168 |
| Chr1_Flank |  |  |  |  | 0.1528 | 2.9019 | 1.5351 | 591 | 11225 | 5.2650 |
| Chr2_Inv | 9735339 | 11326592 | 10786613 | 11092712 | 0.5290 | 3.4174 | 5.7800 | 23305 | 150544 | 15.4805 |
| Chr2_Flank |  |  |  |  | 0.3684 | 3.8631 | 3.8752 | 738 | 7738 | 9.5373 |
| Chr3_Inv | 13237173 | 15315057 | 13267119 | 14856721 | 0.5342 | 3.0672 | 2.784 | 27036 | 155236 | 17.4161 |
| Chr3_Flank |  |  |  |  | 0.0813 | 1.1753 | 0.3295 | 485 | 7012 | 6.9167 |
| Chr4_Inv | 29944680 | 31497199 | 29945905 | 31083844 | 0.3740 | 3.1384 | 2.1232 | 23284 | 195364 | 11.9183 |
| Chr4_Flank |  |  |  |  | 0.0432 | 1.2114 | 0.2683 | 175 | 4900 | 3.5714 |
| Chr5_Inv | 31846446 | 33367531 | 31879615 | 33178717 | 0.4137 | 2.8370 | 2.2784 | 22068 | 151321 | 14.5836 |
| Chr5_Flank |  |  |  |  | 0.0708 | 1.1502 | 0.2953 | 466 | 7569 | 6.1567 |
| Chr6_Inv | 4054500 | 5843062 | 5113931 | 5297292 | 0.3240 | 2.6599 | 1.8979 | 17776 | 145924 | 12.1817 |
| Chr6_Flank |  |  |  |  | 0.0581 | 1.1717 | 0.2825 | 431 | 8692 | 4.9586 |
| Chr7_Inv | 6974687 | 8928400 | 6976451 | 7506754 | 0.3953 | 2.8252 | 3.3850 | 19260 | 137641 | 13.9929 |
| Chr7_Flank |  |  |  |  | 0.0662 | 1.0528 | 0.2628 | 625 | 9941 | 6.2871 |
| Chr9_Inv | 30204250 | 31010400 | 30203078 | 31007705 | 0.2872 | 2.6171 | 1.3572 | 12985 | 118336 | 10.973 |
| Chr9_Flank |  |  |  |  | 0.0295 | 1.0692 | 0.1825 | 390 | 14130 | 2.7601 |
| Chr10_Inv | 8468435 | 9616400 | 8641353 | 9177566 | 0.3284 | 3.3020 | 1.9238 | 11907 | 119716 | 9.9460 |
| Chr10_Flank | |  |  |  | 0.0290 | 1.1765 | 0.2080 | 340 | 13786 | 2.4663 |
| Chr11_Inv | 7116193 | 7834793 | 7217182 | 7502120 | 0.5400 | 3.7423 | 2.1827 | 16098 | 111556 | 14.4304 |
| Chr11_Flank | |  |  |  | 0.1728 | 3.4535 | 1.6222 | 807 | 16130 | 5.0031 |
| Chr12_Inv | 10349492 | 11011892 | 10368123 | 10963525 | 0.5673 | 3.9469 | 3.5855 | 18318 | 127449 | 14.3728 |
| Chr12_Flank | |  |  |  | 0.0121 | 1.0504 | 0.1146 | 139 | 12037 | 1.1548 |
| Chr14_Inv | 26598746 | 28338960 | 26812426 | 28034358 | 0.5176 | 3.2809 | 4.3616 | 30127 | 190969 | 15.7759 |
| Chr14_Flank | |  |  |  | 0.1445 | 1.8147 | 1.0596 | 448 | 5625 | 7.9644 |
| Chr15.1_Inv | 4662067 | 7074518 | 4660382 | 5140287 | 0.6641 | 2.9944 | 3.5791 | 31023 | 139876 | 22.1789 |
| Chr15.1_Flank | |  |  |  | 0.1878 | 2.6786 | 1.5685 | 669 | 9540 | 7.0126 |
| Chr15.2_Inv | 7548498 | 9092262 | 7548218 | 8711215 | 0.3439 | 2.2906 | 2.3342 | 17664 | 117649 | 15.0142 |
| Chr15.2_Flank | |  |  |  | 0.1528 | 1.8096 | 1.2672 | 1213 | 14365 | 8.4441 |
| Chr16_Inv | 28878126 | 31497600 | 29771465 | 31491123 | 0.4707 | 2.3434 | 5.2524 | 27947 | 139129 | 20.0871 |
| Chr16_Flank | |  |  |  | 0.1237 | 1.482 | 0.7369 | 807 | 9665 | 8.3497 |
| Chr17_Inv | 7967707 | 10259765 | 9476120 | 10431338 | 0.6028 | 3.0678 | 7.0178 | 24761 | 126025 | 19.6477 |
| Chr17_Flank | |  |  |  | 0.2304 | 2.0939 | 1.3461 | 1363 | 12385 | 11.0052 |
| Chr18.1_Inv | 0 | 389531 | 75277 | 384089 | 0.8978 | 5.0371 | 2.9558 | 30698 | 172225 | 17.8244 |
| Chr18.1_Flank | |  |  |  | 0.0405 | 2.5743 | 0.4744 | 148 | 9409 | 1.5730 |
| Chr18.2_Inv | 389531 | 1558239 | 506826 | 1111833 | 0.5779 | 3.3164 | 2.6368 | 18180 | 104329 | 17.4256 |
| Chr18.2_Flank | |  |  |  | 0.1474 | 2.4719 | 1.2835 | 1087 | 18225 | 5.9643 |
| Chr20_Inv | 0 | 1125715 | 376265 | 1122469 | 0.4822 | 3.9596 | 2.3662 | 22205 | 182329 | 12.1785 |
| Chr20_Flank | |  |  |  | 0.0332 | 1.0435 | 0.1875 | 230 | 7225 | 3.1834 |
| Chr21_Inv | 24303491 | 25606000 | 24147122 | 24750048 | 0.6648 | 4.7229 | 6.3275 | 22409 | 159201 | 14.0759 |
| Chr21_Flank | |  |  |  | 0.0520 | 1.1204 | 0.2539 | 299 | 6445 | 4.6393 |
| Chr22_Inv | 0 | 899276 | 14626 | 1477166 | 0.4209 | 4.0762 | 2.1993 | 17275 | 167281 | 10.3269 |
| Chr21_Flank | |  |  |  | 0.0287 | 1.2358 | 0.2271 | 246 | 10609 | 2.3188 |
| Chr23_Inv | 12485358 | 13520800 | 12482925 | 13258441 | 0.5339 | 3.4203 | 2.5881 | 20910 | 133956 | 15.6096 |
| Chr23_Flank | |  |  |  | 0.0419 | 1.0412 | 0.2093 | 437 | 10858 | 4.0246 |

*****Calculated based on all windows
