# Supplementary material for: Ancient inversion polymorphisms associate with sexually selected traits across natural guppy populations
Source: PLoS Biol. 2026 Sep 23;24(9):e3004024. doi: 10.1371/journal.pbio.3004024 (PMC13626464; doi:10.1371/journal.pbio.3004024)
Supplement: Supplementary file 2 [file pbio.3004024.s037.docx]

**S2 Table.** Genotype-by-environment test on convergent haplotype differing between high- and low-predation populations.

| SV on Chromosome | overall_*P* | Bonferroni |
| --- | --- | --- |
| 7 | 9.13 × 10⁻³ | 1.37 × 10⁻² |
| 11 | 1.27 × 10⁻^8^ | 5.07 × 10⁻^8^ |
| 21 | 6.86 × 10⁻^6^ | 1.65 × 10⁻^5^ |
