# Supplementary material for: Ancient inversion polymorphisms associate with sexually selected traits across natural guppy populations
Source: PLoS Biol. 2026 Sep 23;24(9):e3004024. doi: 10.1371/journal.pbio.3004024 (PMC13626464; doi:10.1371/journal.pbio.3004024)
Supplement: Supplementary file 3 [file pbio.3004024.s038.docx]

**S3 Table.** Estimated age of inversions in millions of years**.**

| Inversion on Chromosome | Rivers | Age | 95%_Lower | 95%_Upper |
| --- | --- | --- | --- | --- |
| 1 | All | 2.4209 | 2.2298 | 2.6187 |
| 2 | All | 1.5861 | 1.1894 | 2.0391 |
| 3 | All | 2.7816 | 2.6154 | 2.9489 |
| 4 | A, Y | 2.6538 | 2.4223 | 2.8893 |
| 5 | All | 6.1243 | 5.7503 | 6.5009 |
| 6 | A | 3.7538 | 3.1331 | 4.3856 |
| 7 | All | 4.7636 | 4.2144 | 5.3188 |
| 9 | A,Y | 4.2128 | 3.8441 | 4.5958 |
| 10 | All | 4.0498 | 3.6497 | 4.4771 |
| 11 | All | 2.5898 | 2.3125 | 2.8724 |
| 12 | Q,Y | 3.0577 | 2.7956 | 3.3267 |
| 14 | A, Y | 2.3143 | 2.1429 | 2.4815 |
| 15.1 | A, Y | 3.8558 | 3.5135 | 4.1906 |
| 15.2 | All | 1.2170 | 1.1410 | 1.2957 |
| 16 | Y | 2.7492 | 2.6076 | 2.8960 |
| 17 | Y | 2.8150 | 2.6081 | 3.0261 |
| 18.1 | Q,Y | 1.8899 | 1.8487 | 1.9331 |
| 18.2 | All | 3.9241 | 3.5958 | 4.2443 |
| 20 | Q,Y | 3.6459 | 3.3524 | 3.9330 |
| 21 | All | 2.4656 | 2.1147 | 2.8561 |
| 22 | Y | 2.0204 | 1.7958 | 2.2608 |
| 23 | A, Y | 2.6585 | 2.4115 | 2.9028 |
