# Supplementary material for: Ancient inversion polymorphisms associate with sexually selected traits across natural guppy populations
Source: PLoS Biol. 2026 Sep 23;24(9):e3004024. doi: 10.1371/journal.pbio.3004024 (PMC13626464; doi:10.1371/journal.pbio.3004024)
Supplement: Supplementary file 4 [file pbio.3004024.s039.docx]

**S4 Table. Male-to-female read depth ratio of inversions.**

| Inversion on Chromosome | M:F read depth ratio | 95%_Lower | 95%_Upper |
| --- | --- | --- | --- |
| 1 | 1.0010 | 0.9883 | 1.0123 |
| 2 | 1.0109 | 0.9717 | 1.0497 |
| 3 | 0.9884 | 0.9805 | 1.0175 |
| 4 | 1.0033 | 0.9866 | 1.0235 |
| 5 | 0.9767 | 0.9624 | 0.9898 |
| 6 | 1.0229 | 1.0023 | 1.0310 |
| 7 | 1.0258 | 1.0147 | 1.0421 |
| 9 | 0.9990 | 0.9765 | 1.0284 |
| 10 | 1.0082 | 0.9940 | 1.0195 |
| 11 | 1.0080 | 0.9834 | 1.0356 |
| 12 | 1.0204 | 0.9915 | 1.0611 |
| 14 | 1.0070 | 0.9821 | 1.0251 |
| 15.1 | 1.0182 | 1.0365 | 1.0365 |
| 15.2 | 1.0353 | 1.0196 | 1.0597 |
| 16 | 1.0235 | 0.9952 | 1.0718 |
| 17 | 1.0043 | 0.9821 | 1.0200 |
| 18.1 | 1.0459 | 1.0367 | 1.0529 |
| 18.2 | 1.0195 | 1.0019 | 1.0354 |
| 20 | 1.0024 | 0.9904 | 1.0150 |
| 21 | 1.0064 | 0.9902 | 1.0179 |
| 22 | 1.0164 | 0.9967 | 1.0434 |
| 23 | 1.0242 | 0.9984 | 1.0576 |
