# Supplementary material for: Ancient inversion polymorphisms associate with sexually selected traits across natural guppy populations
Source: PLoS Biol. 2026 Sep 23;24(9):e3004024. doi: 10.1371/journal.pbio.3004024 (PMC13626464; doi:10.1371/journal.pbio.3004024)
Supplement: Supplementary file 5 [file pbio.3004024.s040.docx]

**S5 Table. Number of LD-clumped hits within each inversion.**

| INV | pattern space | presence/absence |
| --- | --- | --- |
| Chr1 | 0 | 1 |
| Chr2 | 0 | 0 |
| Chr3 | 2 | 1 |
| Chr4 | 0 | 1 |
| Chr5 | 0 | 0 |
| Chr6 | 0 | 0 |
| Chr7 | 1 | 1 |
| Chr9 | 0 | 0 |
| Chr10 | 0 | 1 |
| Chr11 | 1 | 4 |
| Chr12 | 1 | 2 |
| Chr14 | 2 | 0 |
| Chr15.1 | 2 | 2 |
| Chr15.2 | 2 | 1 |
| Chr16 | 2 | 0 |
| Chr17 | 0 | 0 |
| Chr18.1 | 1 | 0 |
| Chr18.2 | 1 | 1 |
| Chr20 | 0 | 1 |
| Chr21 | 0 | 1 |
| Chr22 | 0 | 0 |
| Chr23 | 0 | 0 |
